# "Vegetative propagation – knowhow and technology for enhancing bioeconomy “ - A new project launched in Finland

**DOI:** 10.1186/1753-6561-5-S7-P142

**Published:** 2011-09-13

**Authors:** Tuija Aronen, Martti Venäläinen, Anni Harju, Teijo Nikkanen

**Affiliations:** 1 Finnish Forest Research Institute, Punkaharju, Finland

## 

A three-year project, financed by EU, European Regional Developmental Fund, will be carried out in 2011-14 as a collaborative effort with Finnish Forest Research Institute as a main performer, and Eastern Finland University and a commercial company Taimityllilä Oy as partners. Three new researchers are engaged by the project. The aim of the project is to deepen knowhow and develop technology for vegetative propagation of forest trees in Finland, and to enhance collaboration among research institutions and practical plant producers. The project strengthens infrastructure for research and development, and benefits industrial and commercial activities in the field. A target group of the project includes forest owners, forest and ornamental nurseries, professionals in landscaping and home gardeners, wood product industry, and research sector. The objectives of the project are divided into five theme areas:

## New solutions for producing forest regeneration material

The main aim of the project is to develop propagation methods for Nordic conifers, especially Norway spruce with a goal set for commercial mass-propagation. This will be done in collaboration with practical plant producers. Tissue culture approach using somatic embryogenesis, potentially combined with cutting technology, will be studied. Also issues related to user rights and royalty fees when using selected clones or bred families will be touched.

## Production of special forms of forest trees for ornamental use and landscaping

Methods of vegetative propagation for special forms of forest trees are developed to meet the increasing demand of hardy conifers for ornamental purposes and landscaping in Northern conditions. Both cutting and tissue culture technology will be tested, and commercial plant production piloted together with a company partner. User right and royalty issues for both natural mutants found or special forms created by breeding are also covered.

## Potentials of vegetative propagation in improving the quality of Scots pine heartwood

The success of tissue culture propagation of Scots pine families producing abundantly extractives, and thus resistant to wood decay, will be compared with the success of families producing only small amounts of extractives. Furthermore, analyses of secondary compounds induced in tissue cultures will be carried out and their use in selection *in vitro* will be studied.

## In vitro –testing of wood quality using mould and decay fungi

Methods of *in vitro* –testing of wood decay resistance will be developed and used to enhance the selection of materials more resistant against fungal degradation. Better decay resistance leads to longer service life of wooden constructions and thus improves the environmental performance of wood.

## Vegetatively propagated materials in enhancing research activities

Clonally propagated materials would greatly benefit e.g. pathological and entomological studies in Nordic conifers. In addition, developed technologies and knowhow can be utilised in forest tree breeding programmes by adopting clonal testing options.

